# MULTIPROFESSIONAL ELECTRONIC PROTOCOL FOR DIGESTIVE SURGERY VALIDATION

**DOI:** 10.1590/0102-672020210002e1583

**Published:** 2021-10-18

**Authors:** Faruk Abrão KALIL-FILHO, José Simão de Paula PINTO, Emerson P BORSATO, Carlos Henrique KURETZKI, Bruno Luiz ARIEDE, Jorge Eduardo Fouto MATHIAS, Antonio Carlos Ligocki CAMPOS, Osvaldo MALAFAIA

**Affiliations:** 1 Postgraduate Program in Surgical Clinic, Health Sciences Sector, Federal University of Paraná, Curitiba, PR, Brazil; 2 Postgraduate Program in Informatics, Exact Sciences Sector, Federal University of Paraná, Curitiba PR, Brazil; 3 Biomedical Informatics, University of Utah, Salt Lake City, Utah, USA; 4 Mackenzie Evangelical College of Paraná, Curitiba, PR, Brazil; 5 Positivo University, Curitiba, PR, Brazil

**Keywords:** Medical Informatics Applications, Protocols, Data collection., Aplicações da Informática Médica, Protocolo, Coleta de dados

## Abstract

***Background*:**

The creation of a computerized clinical database with the ability to collect prospective information from patients and with the possibility of rescue and crossing data enables scientific studies of higher quality and credibility in less time.

***Aim*:**

To validate, in a single master protocol, the clinical data referring to Surgery of Digestive System in a multidisciplinary way, incorporating in the SINPE^©^ platform, and to verify the incidence of digestive diseases based on the prospectively performed collections.

***Method*:**

Organize in one software, in a standardized structure, all the pre-existing items in the SINPE^©^ database; the theoretical basis was computerized through the MIGRASINPE^©^ module creating a single multiprofessional master protocol for use as a whole.

***Results*:**

The existing specific protocols were created and/or adapted - they correspond to the most prevalent digestive diseases - unifying them. The possibility of multiprofessional use was created by integrating all data collected from medicine, nursing, physiotherapy, nutrition and health management in a prospective way. The total was 4,281 collections, distributed as follows: extrahepatic biliary tract, n=1,786; esophagus, n=1015; anorectal, n=736; colon, n=550; small intestine, n=86; pancreas, n=71; stomach, n=23; liver, n=14.

***Conclusions*:**

The validation of the unification and structuring in a single master protocol of the clinical data referring to the Surgery of the Digestive System in a multiprofessional and prospective way was possible and the epidemiological study carried out allowed to identify the most prevalent digestive diseases.

## RESUMO

**Racional:**

A criação de um banco de dados clínicos informatizado com a capacidade de coletar informações dos pacientes de forma prospectiva e com possibilidade de resgate e cruzamento viabiliza estudos científicos de maior qualidade e credibilidade em menor tempo.

**Objetivos:**

Validar em único protocolo mestre os dados clínicos referentes à Cirurgia do Aparelho Digestivo de forma multiprofissional incorporando-o na plataforma SINPE^©^, e verificar a incidência das doenças digestivas com base nas coletas prospectivamente realizadas_._

***Método*:**

Organizar no software em estrutura padronizada todos os itens pré-existentes no banco de dados do SINPE^©^, informatizar a base teórica através do módulo MIGRASINPE^©^ criando-se um único protocolo mestre multiprofissional para uso como um todo.

**Resultados:**

Foram criados e/ou adaptados os protocolos específicos existentes que correspondem às doenças digestivas mais prevalentes unificando-os. Criou-se a possibilidade de uso multiprofissional integrando todos os dados coletados da medicina, enfermagem, fisioterapia, nutrição e gestão em saúde de maneira prospectiva. O total foi de 4.281 coletas assim distribuídas: vias biliares extra-hepáticas, n=1.786; esôfago, n=1015; anorretais, n=736; cólon, n=550; intestino delgado, n=86; pâncreas, n=71; estômago, n=23; fígado, n=14.

**Conclusões:**

A validação da unificação e estruturação em único protocolo mestre dos dados clínicos referentes à Cirurgia do Aparelho Digestivo de forma multiprofissional e prospectiva foi possível e o estudo epidemiológico realizado permitiu identificar as doenças mais prevalentes nesse aparelho.

## INTRODUCTION

The production of clinical or experimental studies is the basis for the development of any area of ​​medical knowledge. Thus, the evolution of Medicine is directly linked to the production of quality literature. The integration between Informatics and Medicine has been decisive in the medical literature. The use of computer resources, especially in regard to the capture, storage and search of clinical data, has been of great importance in the production of relevant and reliable clinical studies^1^ ^,^ ^4^ ^,^ ^6^ ^,^ ^8^.

The creation of an electronic database of clinical and surgical data, in research centers, based on the use of electronic protocols, allows a great capacity for information storage and processing. It also facilitates data access and recovery, allowing prospective, high-quality scientific work to be carried out in shorter time^11^.

There are several studies being carried out in several institutions for the use of health protocol systems^12^. They usually show results showing that the use of protocols in medical practice helps health professionals during the care process, minimizing errors, standardizing the services provided and increasing quality^5^.

Protocol is a cognitive model that represents a facet of medical knowledge applied to a particular purpose of data collection^7^. It is used to standardize and standardize data collection in a health institution, reason to frequent use of data collection forms. Thus, the development of clinical electronic protocols capable of carrying out prospective structured storage is a very useful tool in the production of quality medical literature.

The creation of a computerized clinical database with the ability to collect clinical information prospectively and with the possibility of retrieving and crossing the data, enables clinical research with a high level of reliability. The unification of these clinical data in a single master protocol would provide standardization for future multiprofessional protocols, interaction in different areas of health, better search for information with better analysis of the data and possibility of cross-referencing the data in a multiprofessional way.

For that, the SINPE^©^ (Integrated System of Electronic Protocols) computer program created by Prof. Dr. Osvaldo Malafaia - who owns his intellectual property - registered with the National Institute of Industrial Property - INPI of the Ministry of Industry, Foreign Trade and Services of Brazil as a Computer Program Registry no. RS 06056-1 on February 17, 2009.

**FIGURE 1:**
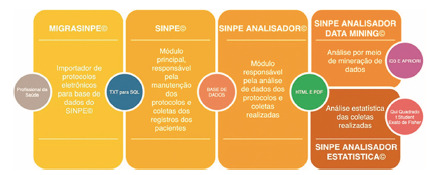
Modules that make up the SINPE^©^ system of electronic protocols

SINPE^©^ is composed of four modules, represented in Figure 1

SINPE^©^ also allows data to be collected in a multicenter environment, storing it in a central database. In this way, it makes possible to conduct multi-center prospective research online.

Thus, the objectives of this work were to validate the clinical data referring to Digestive System Surgery in a multiprofessional way in a single master protocol, incorporating it in the SINPE^©^ platform, and to verify the incidence of digestive diseases based on the prospectively performed collections.

## METHOD

This is a descriptive study with methodology divided into four phases: 1) unification and structuring of the master protocol of clinical data referring to Surgery of the Digestive System in a multidisciplinary way; 2) computerize the theoretical database through SINPE^©^; 3) implant the base in the “master protocol” and making/adapting specific protocols; 4) to analyze the incidence of diseases of the digestive system in a tertiary referral university hospital.

### Unification and structuring of the master protocol of clinical data related to Digestive System Surgery in a multidisciplinary way

For the realization of the new concept of standard structure in the program, it was necessary to reorganize in a didactic way all the items existing in the SINPE^©^ database. This bank had a total of 49,996 items, all related to the protocols created for digestive surgery. The existing protocols belonged to a single database, but each segment of the digestive system was separated, each by a master protocol. Eight computerized protocols existing in SINPE^©^ were used for this structuring, namely: diseases of the esophagus, stomach, small intestine, colon, rectum and anus, liver, extrahepatic bile ducts and pancreas. Each master protocol already had its respective specific protocols representing the diseases of the anatomical segment in question, making a total of 136 protocols.

To initiate the reorganization of information and the creation of the multiprofessional protocol, it was structured in Word^©^ format. The new structure was based on a didactic format, starting with anamnesis, physical examination, complementary exams, diagnosis, treatment and evolution. They form the main root of the master protocol. The first three items in the main root have items that are common to all specialties. The items diagnosis, treatment and evolution have particularities for each profession, and were thus separated. The first stage was summarized in bringing together the thematic items (as an example, aggregating all the anamnesis items from the eight protocols into a single anamnesis item), eliminating the ones that presented duplicity and adding other related health areas, that is, Nursing, Physiotherapy, Nutrition and Health Management.

Computerization of the theoretical database through SINPE^©^

**FIGURE 2:**
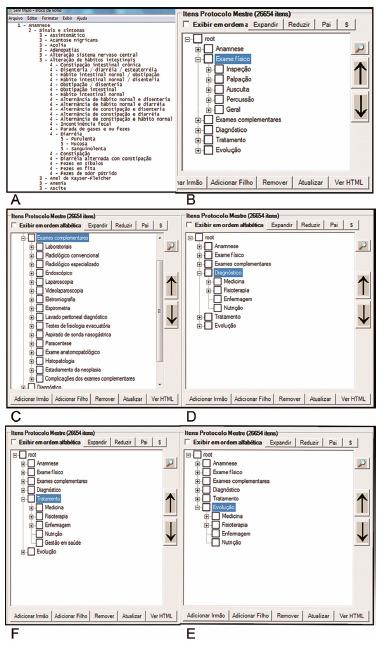
Examples of data collection: A) anamnesis; B) physical examination; C) complementary exams; D) diagnosis; E) treatment; F) evolution.

To assist in this process, a module from the SINPE^©^ software, MIGRASINPE^©^, was used, capable of automatically importing the theoretical database to SINPE^©^, generating the complete master protocol, without losing the original form of its hierarchy and automatically creating a new one database. By importing a text file that contains the hierarchically grouped data, MIGRASINPE^©^ inserts the data into the Microsoft Access^©^ database, making this information available. Figure 2 shows examples of the use of this module.

### Implement the base on the “master protocol” and making/adapting “specific protocols”

All items of the “Master Protocol” (26,634 items) of Surgery of the Digestive System were computerized and incorporated into SINPE^©^, allowing access of this information either locally or remotely, via internet.

### To analyze the incidence of diseases of the digestive system in a tertiary referral university hospital.

Prospective data collection was performed with the SINPE^©^ software at the Digestive System Surgery Service at Hospital das Clínicas, Federal University of Paraná, Curitiba, PR, Brazil, using the existing database, but the master protocols divided, without current format and without multiprofessionality. Next, data were prospectively collected from patients who underwent surgical treatment at the service. In the new structuring in a multiprofessional protocol, now presented, the previous collections were incorporated, which were inserted without modifications in the new protocol, manually.

In recent years, data collection has resumed, but with the new Electronic Multiprofessional Protocol for Digestive System Surgery, forming a total of 4281 collections. They were carried out with totally prospective information, and not only obtained from medical records, but also from the patients themselves at the bedside, in order to minimize collection errors and make the data more reliable.

For analysis, the SINPE ANALYZER^©^ module was used, an interface for displaying information from SINPE^©^ in order to generate graphs, statistics, print, save results and export data, providing quality information easy to interpret. After selection, this module allows obtaining data regarding the base, date of creation, number of items, authors of collections, places of collection, number of patients by gender and race and their combinations, frequency distribution of patients as to the range age, items collected and not collected, with textual presentations, in table and graphs.

## RESULTS

The results of this research will be presented by figures corresponding to the computer screens in a summarized mode. For a better monitoring and full visualization of the program, the reader will be able to carry out, simultaneously with this reading, a video visualization of the handling of the software that shows all the steps of the navigation. This tutorial is attached to this article and can be accessed by link https://youtu.be/0eOR57zWcUM or by the QRcode at the beginning of this publication.

### Multiprofessional master protocol for digestive tract surgery.

**FIGURE 3:**
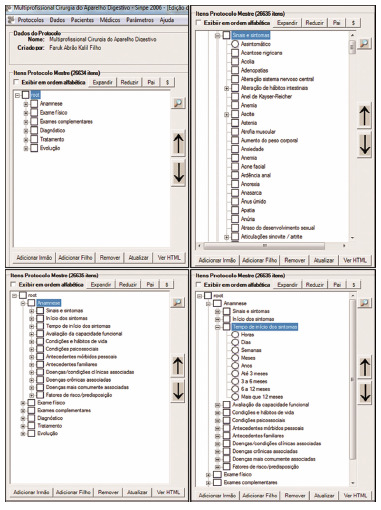
Examples of detailed data insertion in the protocols

The main screen of SINPE^©^ displays the menu bar “Protocols”, “Data”, “Patients”, “Doctors”, “Parameters” and “Help”. By clicking on “Protocols”, the options “Master” and “Specific” and “Exit” appear on the screen. As an example, Figure 3 presents the master protocol and specific esophageal diseases.

The anamnesis item was created by compiling all data in the protocols. It starts with “Signs and symptoms” and ends with “Risk/predisposition factors”. It has a total of 3,275 subitems. The items were arranged according to the natural sequence of questions that are asked in medical consultations (Figure 3). Like the example in Figure 3, the other phases of data collection follow the same scheme based on the logical principle of medical care. They are in relation to anamnesis: Conditions and Habits of Life; Personal Morbid Background; Family History; Associated Diseases/Clinical Conditions; Risk/Predisposition Factors.

In the same way exemplified in the Anamnesis (Figure 3), the other items - always with the characteristic of collecting items arranged hierarchically - follow the Physical Examination, Complementary Examinations, Diagnosis, Treatment and Evolution.

**FIGURE 4:**
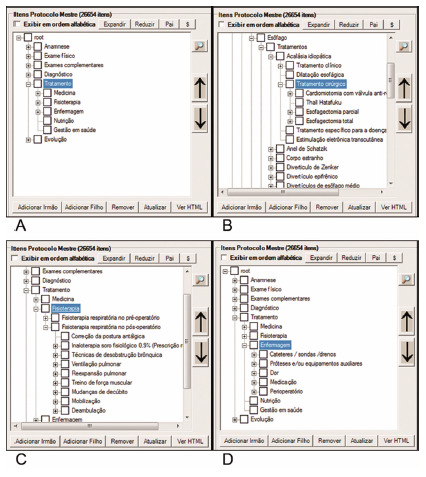
Examples of multidisciplinary sub-items of the item Treatment: A) choose in the root the desired item, that was treatment in Medicine; B) choose between the possible treatments in the esophagus; C) choose treatments applicable to physiotherapy in the same patient; D) same conditions in nursing.

For use in its multiprofessional characteristic, in aspects where the specialties have different collections, icons are opened to choose the specialty and desired details (Figure 4).

**FIGURE 5:**
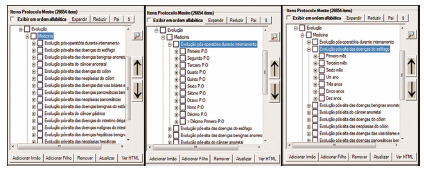
Example of postoperative follow-up

Emphasis should be given to the postoperative follow-up, here called Evolution. Figure 5 shows an example in Medicine, but the other professions follow the same sequence. This item was created to carry out prospective data collection and presents the patient’s evolution from the first postoperative day, containing information such as complications, analogue pain scale, aspect of the surgical scar, among other important postoperative items (Figure 5).

### Creation of a specific protocol according to the researcher’s desire in his daily clinic

In addition to the protocols already created and which are stored in SINPE^©^, any others can be created at the wish of the attending physician and who wishes to do individual research. The specific protocols were previously registered so that it was possible to make them in this program. They were reorganized using the data contained in the master protocol, obeying the same order of disposition in which they were created by other authors. Only the structure of the main root was changed, without losing the reliability of the previous protocols. All 136 specific protocols previously created were registered (Figure 5).

Analysis of the incidence of diseases of the digestive system based on the 4,281 prospective collections performed at SINPE^©^

For better understanding, the exposure of the results will be divided into: Compilation of the results and Collections by computerized protocol.

#### Compilation of results

In 40 months, 4,281 prospective data collections were carried out in computerized protocols related to diseases of the digestive system.

 SINPE^©^ allows the same patient to be collected more than once, but in another specific protocol. In this collection there are patients who were collected more than once; as an example, one who underwent cholecystectomy and later operated on hiatus hernia. He has only a single record, but with more than one collection.

In the general collection of this study, the total number of registered patients was 3,870 with 4,281 collections in specific protocols, with 411 patients having undergone more than one surgical procedure.

Of the 3,870 patients, 2,280 were women (58.91%) and 1,590 men (41.09%). The electronic database with the largest number of collections was on diseases of the extrahepatic biliary tract with 1,786 collections, followed by those of the esophagus with 1015, anorectal with 736, colon with 550, small intestine with 86, pancreas with 71, stomach with 23 and the liver with 14 collections. The specific protocol for chronic lithiasic cholecystitis was the one that obtained the largest number of collections (1583), followed by gastroesophageal reflux disease (900).

#### Collections by computerized protocol

Figure 7 shows the incidence of various diseases in the eight digestive segments analyzed in SINPE^©^.

**FIGURE 6:**
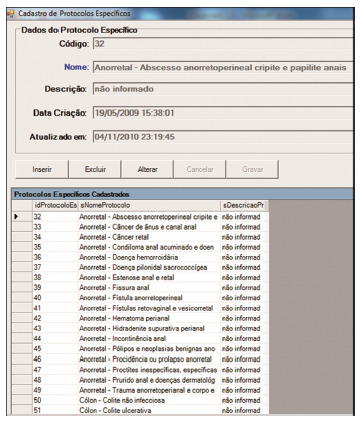
Examples of specific protocols created by coloproctologists in their clinics

**FIGURE 7:**
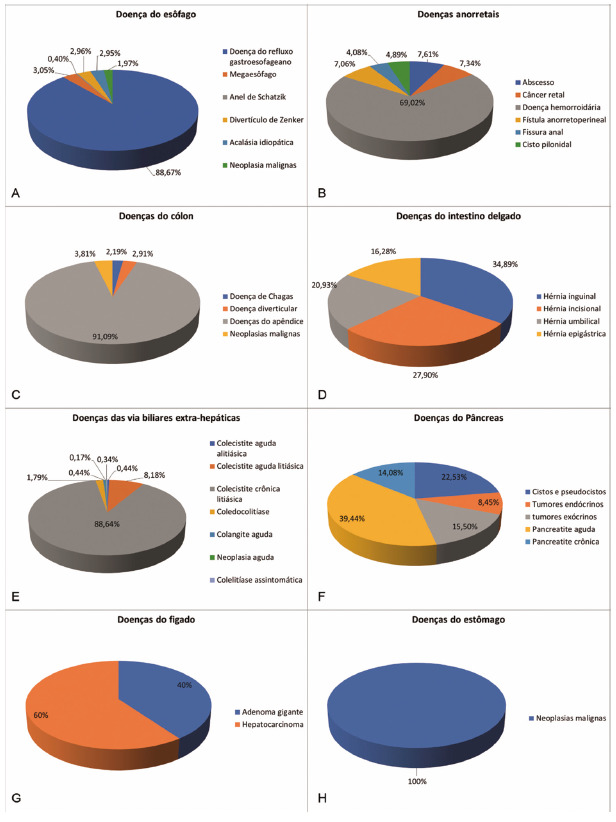
Incidences of the various diseases found in the included eight digestive segments/organs

The esophageal base consisted of 27 specific protocols and had 1015 collections, corresponding to 23.71% of all collections performed (Figure 7A). The anorectal disease protocol consisted of 18 specific protocols and presented 736 collections (17.20%, Figure 7B); in the case of colon diseases, there were 16 with 550 collections (12.85%, Figure 7C); in diseases of the small intestine there were 24 with 86 collections (2.01%, Figure 7D); in the extrahepatic bile ducts there were 25 with 1786 collections (41.72%, Figure 7E); in pancreatic diseases, there were five with 71 collections (1.66%, Figure 7F); in diseases of the eight were with 14 collections (0.32%, Figure 7G); in stomach diseases, there were 13 with only 23 malignant neoplasms. This last data is justified due to the fact that most of the related diseases are of non-operative treatment and this protocol refers to the operations performed (7H).

## DISCUSSION

### Health informatics

Information is the element that supports every exercise of health care practice. Currently, technology brings benefits as it allows information to be transformed into scientific knowledge quickly and safely, and information technology has become indispensable in helping researchers in the search for scientific quality in their work.

Information and communication technologies have made it possible to change the characteristics of the practice of Medicine and related areas, as they allow the large volume of information that is generated continuously to be made available^6^ ^,^ ^8^. Ferreira (1996)^9^ points out that “Systems must be modeled according to users, with the nature of their information needs and with their behavioral patterns in the search and use of information”.

Health informatics, in addition to data storage, also includes the production of studies with large series of patients containing reliable information; consequently, the production of meta-analyzes and guidelines has produced good results. Recent studies demonstrate that these new technologies are an important part of improving the treatment of patients with chronic diseases, corroborating the importance of structured collection of clinical data^2^ ^,^ ^15^.

The quality of the data obtained from the electronic protocols is more complete, has few errors, is more consistent and has a low percentage of violation in relation to the medical records on paper. The processes of editing, typing, checking and clarifying can be eliminated, and irrelevant questions can be omitted^7^.

About the preparation of the electronic protocol and its incorporation to SINPE^©^

The initial idea of ​​this work was to group all the electronic protocols developed in surgery of the digestive system in a single master protocol. The master protocols were divided into the SINPE^©^ database; as an analogy, it would be like an octopus, where each tentacle would represent an organ/segment of the digestive system and the head would be the great repository receiving and storing all the information contained in the tentacles in order to integrate and interact with them.

As SINPE^©^ already presented protocols created in other health areas - Physiotherapy, Nursing, Nutrition and Management - and with the need to change the formatting of the protocols, the idea was used to add multiprofessionality to the program. The demanded health needs and the growing development scientific and technological have produced strategies and mechanisms to make teamwork effective, with quality and efficiency. This work requires specificity from each professional and common areas based on practices and knowledge that are common to everyone. The multiprofessional team is, today, a necessary reality in all spaces where actions are taken to improve the quality of health and life of the populations. The Multiprofessional Electronic Protocol for Digestive Surgery was, then, created and the items were reorganized to incorporate specific protocols. The next protocols to be created will follow the same structure, standardizing the format of the program, not being allowed to change its main structure, thus maintaining this new concept.

The idea of ​​including all protocols in a single master protocol found the inconvenience of the large number of diseases and the enormous number of items added up, presenting greater difficulty in reorganizing the items, having to separate and eliminate those with duplication. Another difficulty was making it practical in view of the complexity of the protocols and trying to structure it in the best possible way to keep it as reliable as possible. The structuring followed didactic order, being the main root unique and unalterable where the items that belong to this root are the same. The items anamnesis, physical exam and complementary exams are common to all professions. Diagnosis, treatment and evolution are different items in the various professions, and so they were separated. The development of a single multiprofessional database allows its use in scientific studies with more reliable conclusions, both retrospective and prospective.

For the implementation of the protocol to be successful and accepted, it needs reliable evaluation by independent professional groups. Users need to be involved in priority levels and planning for updated implementations^14^. The program is constantly undergoing updating processes, as it must meet the requirements of users and be up to date with advances in technology.

The project called “Electronic Protocols” has been improved since 1999, with constant updates and program improvements, with inclusions such as SINPE^©^ MULTIPROFESSIONAL, MIGRASINPE^©^, SINPE^©^ ANALYZER, SINPE^©^ ANALYZER DATAMINING, and SINPE^©^ STATISTICAL ANALYZER. Currently, there are multiprofessional protocols created in traumatology, ophthalmology, vascular surgery and cardiac surgery, neurology and others, with a large amount of data. The objective of SINPE^©^ has always been to seek information from the patient and also from his medical record, collecting information from anamnesis to outpatient follow-up. Allied to different crossings, information will emerge as a source for scientific basis in several prospective studies.

### Epidemiological analysis of collected diseases

In this study, there was no restriction in relation to computerized protocols, as data were not collected on a specific protocol, but prospective data collected on all eight digestive disease protocols registered with SINPE^©^. Then, 4281 cases were included with fully prospective information not only obtained from medical records, but also from the patients themselves at the bedside, in order to minimize collection errors and make the data more reliable.

SINPE^©^’s proposal is to collect data based on the specific protocol, that is, based on the diagnosis. Thus, the distribution of cases was able to demonstrate the main types of diseases that occurred in the studied period.

Further research will be carried out from this bank with a significant number of cases collected and a huge number of items to be analyzed. These data can now be crossed, mined and, after statistical analysis incorporated into the program itself, generate high quality scientific studies.

## CONCLUSION

It was possible to unify and structure a single master protocol containing all the clinical and surgical data related to Digestive System Surgery in a multidisciplinary way. The computerized database in the software, derived from systematic collection, was carried out in the form of multiprofessional electronic protocols and could be incorporated into SINPE^©^. The resulting epidemiological study can serve as a guide to health actions focusing on the main causes of digestive diseases in our environment.
